# Advantage of laparoscopic surgery in patients with generalized obesity operated for colorectal malignancy: A retrospective cohort study

**DOI:** 10.3389/fsurg.2022.1062746

**Published:** 2023-01-06

**Authors:** Yen-Lin Yu, Yu-Jen Hsu, Chun-Kai Liao, Yueh-Chen Lin, Jeng-Fu You, Wen-Sy Tsai, Bor-Kang Jong, Yih-Jong Chern

**Affiliations:** ^1^ Division of Colon and Rectal Surgery, Department of Surgery, Chang Gung Memorial Hospital, Keelung Branch, Keelung City, Taiwan; ^2^ School of Medicine, Chang Gung University, Taoyuan City, Taiwan; ^3^ Division of Colon and Rectal Surgery, Department of Surgery, Chang Gung Memorial Hospital, Linkou Branch, Taoyuan City, Taiwan

**Keywords:** laparoscopic surgery, obesity, BMI, colorectal cancer, minimal invasive surgery

## Abstract

**Background:**

Because of the progression of minimally invasive surgery skills and obesity in colorectal surgery, we aimed to evaluate the short-term outcomes of colorectal cancer resections in patients with generalized obesity at a single teaching hospital with mature surgical techniques and training programs.

**Methods:**

A total of 537 patients were diagnosed with CRC and had a body mass index ≥30 kg/m^2^ between January 2009 and December 2019 at a single institution. 265 patients underwent open surgery and 272 patients underwent laparoscopic surgery. Data were analysed to explore the independent risk factors for postoperative complications.

**Results:**

The laparoscopic group had less blood loss (73 ± 128 vs. 148 ± 290 ml, *p* < 0.001) and a shorter postoperative hospital stay (10.8 ± 17.1 vs. 11.7 ± 6.8 days, *p* < 0.001) than the open group. The number of harvested lymph nodes did not significantly differ between the two groups (30.9 ± 18.3 vs. 30.2 ± 15.3, *p* = 0.981). Although anastomotic leakage was significantly higher in the laparoscopic group (1.5% vs. 4.8%, *p* = 0.030), there were also similar overall postoperative morbidity and mortality rates between the open and laparoscopic groups for CRC patients with generalized obesity who underwent surgery.

**Conclusion:**

Laparoscopic surgery can reduce blood loss, decrease the length of hospital stay, obtain a similar number of harvested lymph nodes, and achieve an acceptable conversion rate for CRC patients with generalized obesity. We suggest that laparoscopic surgery could become a standard method for CRC treatment in patients with generalized obesity.

## Background

Obesity is an increasingly significant public health challenge, particularly in developed countries. The worldwide prevalence of obesity has nearly tripled between 1975 and 2016 (1). According to the World Health Organization standard, generalized obesity was defined as a body mass index (BMI) ≥30 kg/m^2^ (2). Generalized obesity leads to metabolic syndrome and other serious diseases, such as type 2 diabetes mellitus, hypertension, dyslipidaemia, coronary heart disease, ischaemic stroke, gastroesophageal reflux disease, non-alcoholic fatty liver diseases, chronic kidney disease, and several malignancies, including colorectal cancer (CRC) (3, 4). Compared with patients of normal weight, obese patients are likely to have comorbidities that lead to postoperative complications of gastrointestinal tumours (5–7).

The surgical skills of minimally invasive surgery, such as laparoscopic or robotic surgery for CRC, have gradually matured for decades. Many studies have demonstrated that laparoscopic colectomy improved patients’ hospital stay, surgical site complications, and return to routine life (8). The long-term outcomes of 3-, 5-, and even 10-year analyses were also not different between the open and laparoscopic groups (9–13).

Because of the associated comorbidities and increased visceral adiposity, obese patients pose many different challenges during surgery. For the minimally invasive approach, the specific surgical challenges include difficulty in performing high ligation of the mesenteric vessels because of the shortened and bulky mesentery, identification of the correct dissection planes, and difficulty in maintaining the surgical space due to visceral adiposity. Thus, for laparoscopic surgery, obese patients have a longer operative time, greater blood loss, higher blood transfusion requirements, reduced number of harvested lymph nodes, higher conversion rates, increased hospital stay, and complication rates than non-obese patients (14–16).

Because of the rapid progression in minimally invasive surgery skills in colorectal surgery and the increasing number of obese patients worldwide, there is a need to perform an updated analysis of the short-term outcomes of laparoscopic surgery for obese patients. Therefore, the aim of this study was to evaluate the short-term outcomes of laparoscopic and open CRC resections in generalized obese patients at a single teaching hospital.

## Methods

### Study design and population

**Figure 1:**
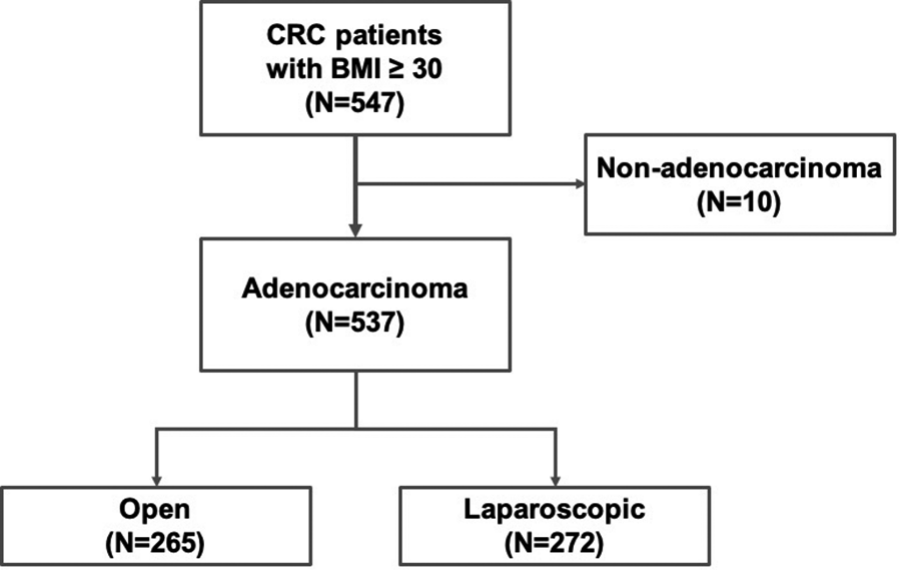
Flow chart of patient screening and selection.

We collected data from patients diagnosed with primary CRC and a BMI ≥30 kg/m^2^ between January 2009 and December 2019 at Chang Gung Memorial Hospital. Data collection and analysis were supervised and approved by the Institutional Review Board of Chang Gung Memorial Hospital in Taiwan (IRB No. 202000644B0). After the radial operation, the pathology reports indicated that non-adenocarcinoma cases would be excluded from the study. A total of 537 patients were enrolled in the analysis. Of the enrolled patients, 265 underwent open surgery and 272 underwent laparoscopic surgery. According to the NCCN guidelines, the decision to perform open or laparoscopic surgery was based on the surgeons’ experience and patients’ performance (Figure 1) (17).

### Data collection

The clinical laboratory test results, operative characteristics, and pathological features were obtained from patients’ electronic medical records and the Colorectal Section Tumor Registry of Chang Gung Memorial Hospital. Patients’ clinical demographic data, including sex, age, and BMI, were recorded. Medical record data, such as the incidences of hypertension, cardiac disease, cerebral vascular disease, asthma, diabetes mellitus, liver cirrhosis, thyroid disease, renal insufficiency, and history of previous abdominal operation and neoadjuvant therapy were collected. The Charlson Comorbidity Index (CCI) was calculated to evaluate the pre-operative comorbidity (18). Preoperative laboratory results, including levels of the carcinoembryonic antigen (CEA), haemoglobin, absolute lymphocyte count, and albumin, were also recorded. Operative features included the tumour location, emergency operation, type of operation, operative time, blood loss, length of postoperative hospital stay, readmission rate, postoperative morbidity and mortality, and conversion rate. Pathological features included the tumour size, depth of tumour invasion (T stage), lymph node involvement (N stage), histologic type and grade, and the number of resected lymph nodes.

### Study endpoints

The endpoints were short term overall and included postoperative morbidity or mortality, the operative time, operative blood loss, duration of postoperative hospital stay, and number of resected lymph nodes. Postoperative morbidity included surgical-related complications (wound infection, ileus, obstruction, gastrointestinal bleeding, intra-abdominal infection, and anastomosis leakage) and postoperative systemic-related complications (pulmonary disease, cardiovascular disease, and urinary tract disease). Postoperative mortality was defined as any death regardless of cause, occurring within 30 days after surgery or during the same hospitalisation subsequent to the operation (19). All the postoperative complications severity were also be classified by Clavien-Dindo classification (20).

### Statistical analysis

Statistical analyses were performed using the Statistical Package for the Social Sciences version 26 (IBM Corp., Armonk, NY, USA). The clinical characteristics were compared using the chi-square test for categorical variables and the Mann-Whitney *U* test for continuous variables. To explore the independent risk factors for postoperative complications, the variables with statistical significance (*p* < 0.1) in the univariate analysis were entered into a multivariate binary logistic regression analysis with backward model selection to identify the independent variables associated with postoperative complications in CRC patients with a BMI ≥30 kg/m^2^. All statistical differences were considered significant at *p* < 0.05.

## Results

### Demographic characteristics

We enrolled and analysed 537 patients and divided them into an open group (265 patients) and a laparoscopic group (272 patients). The clinical characteristics of patients in both groups are shown in Table 1. There were no significant differences in age, sex ratio, BMI, the grading of obesity, and CCI. In terms of comorbidities, patients in the laparoscopic group had higher rates of diabetes mellitus [27.9% versus (vs.) 37.5%, *p* = 0.018] and liver cirrhosis (0.4% vs. 2.6%, *p* = 0.036) than patients in the open group. Patients in the open group had a higher rate of history of abdominal surgery (24.9% vs. 17.3%, *p* = 0.030). The open group showed significantly advanced tumour-node-metastasis stage and a higher rate of abnormal preoperative serum CEA levels (33.3% vs. 23.3%, *p* = 0.011).

**Table 1:** Clinical demographic characteristics of the study patients.

Demographic characteristic	All patients	Open	Laparoscopic	*p*-value
Number of patients (%)	537 (100)	265 (49.3)	272 (50.7)	
Sex (male)	281 (52.3)	124 (46.7)	157 (57.7)	0.011
Age ≥65 years	230 (42.8)	110 (41.5)	120 (44.1)	0.541
BMI (kg/m^2^)	32.43 ± 2.53	32.51 ± 2.47	32.35 ± 2.60	0.512
Obesity grading				0.712
I	472 (87.9)	230 (86.8)	242 (89.0)	
II	60 (11.2)	32 (12.1)	28 (10.3)	
III	5 (0.9)	3 (1.1)	2 (0.7)	
Stage				<0.001
0	8 (1.5)	3 (1.1)	5 (1.8)	
1	125 (23.3)	42 (15.8)	83 (30.5)	
2	154 (28.7)	79 (29.8)	75 (27.6)	
3	191 (35.6)	100 (37.7)	91 (33.5)	
4	59 (11.0)	41 (15.5)	18 (6.6)	
Hypertension	352 (65.5)	171 (64.5)	181 (66.5)	0.623
Cardiac disease	78 (14.5)	33 (12.5)	45 (16.5)	0.242
Cerebral vascular disease	25 (4.7)	12 (4.5)	13 (4.8)	0.890
Asthma	28 (5.2)	17 (6.4)	11 (4.0)	0.217
Diabetes mellitus	176 (32.8)	74 (27.9)	102 (37.5)	0.018
Liver cirrhosis	8 (1.5)	1 (0.4)	7 (2.6)	0.036
Thyroid disease	8 (1.5)	4 (1.5)	4 (1.5)	0.970
Renal insufficiency	74 (13.9)	36 (13.7)	38 (14.0)	0.939
Previous abdominal operation	113 (21.0)	66 (24.9)	47 (17.3)	0.030
Neoadjuvant therapy	51 (9.5)	30 (11.3)	21 (7.7)	0.155
CCI	4.95 ± 2.03	5.02 ± 2.02	4.89 ± 2.04	0.476
CEA level ≥5 ng/ml	149 (28.2)	86 (33.3)	63 (23.3)	0.011
Hb level <10 g/dl	87 (16.2)	40 (15.2)	47 (17.3)	0.504
Absolute lymphocyte count <1,500	122 (23.5)	65 (25.2)	57 (21.8)	0.368
Albumin level <3.5 g/dl	26 (4.9)	14 (5.4)	12 (4.4)	0.582

Data are presented as *n* (%) unless otherwise indicated; the *p*-value was determined by the chi-square test for multiple comparisons and the Mann-Whitney *U* test for continuous variable (BMI and CCI). BMI, body mass index; CCI, Charlson comorbidity index; CEA, carcinoembryonic antigen; Hb, haemoglobin.

### Operative findings

The operative data are shown in Table 2. There was no significant difference in the tumour location, operative method, and rate of emergency surgery between the two groups. The open group had a higher rate of combining other surgeries than the laparoscopic group (11.3% vs. 4.0%, *p* = 0.002). However, the mean operative time was significantly longer in the laparoscopic group than in the open group (244 ± 99 vs. 272 ± 98 min, *p* < 0.001). Compared with the open group, the laparoscopic group had significantly less blood loss (148 ± 290 vs. 73 ± 128 ml, *p* < 0.001) and a significantly shorter postoperative hospital stay (11.7 ± 6.8 vs. 10.8 ± 17.1 days, *p* < 0.001). No significant differences in overall postoperative morbidity and readmission rates found between the two groups. Among the postoperative morbidity parameters, only anastomotic leakage was significantly higher in the laparoscopic group than in the open group (1.5% vs. 4.8%, *p* = 0.030). The postoperative mortality rates were comparable between the open group (0.8%) and the laparoscopic group (1.1%) (*p* = 0.675). And the severity of postoperative complications (Clavien-Dindo classification ≥3) was also the same in the open or laparoscopic group (2.3% vs. 5.5%, *p* = 0.052). The conversion rate in the laparoscopic group was 2.6%.

**Table 2:** Operative characteristics of the study patients.

Operative characteristic	All patients	Open	Laparoscopic	*p*-value
**Number of patients (%)**	537 (100)	265 (49.3)	272 (50.7)	
**Tumour location**				0.311
Right colon	165 (30.7)	73 (27.5)	92 (33.8)	
Left colon	208 (38.7)	105 (39.6)	103 (37.9)	
Rectum	164 (30.5)	87 (32.8)	77 (28.3)	
**Emergency surgery**	6 (1.1)	5 (1.9)	1 (0.4)	0.094
**Type of operation**				0.134
Right hemicolectomy	134 (25.0)	57 (21.5)	77 (28.3)	
Left hemicolectomy	45 (8.4)	23 (8.7)	22 (8.1)	
Anterior resection	321 (59.8)	160 (60.4)	161 (59.2)	
Abdominal perineal resection	7 (1.3)	5 (1.9)	2 (0.7)	
Segmental resection	5 (0.9)	2 (0.8)	3 (1.1)	
Subtotal colectomy	11 (2.0)	7 (2.6)	4 (1.5)	
Hartmann's procedure	14 (2.6)	11 (4.2)	3 (1.1)	
**Combined with other surgery**	41 (7.6)	30 (11.3)	11 (4.0)	0.002
**Operative time (min)**	258 ± 99	244 ± 99	272 ± 98	<0.001
**Blood loss (ml)**	110 ± 226	148 ± 290	73 ± 128	<0.001
**Length of postoperative hospital stay (days)**	11.3 ± 13.1	11.7 ± 6.8	10.8 ± 17.1	<0.001
**Postoperative morbidity**	84 (15.6)	41 (15.5)	43 (15.8)	0.914
Wound infection	27 (5.0)	17 (6.4)	10 (3.7)	0.147
Lung infection	7 (1.3)	4 (1.5)	3 (1.1)	0.678
Cardiovascular event	3 (0.6)	1 (0.4)	2 (0.7)	0.578
Urinary infection	12 (2.2)	7 (2.6)	5 (1.8)	0.529
Gastrointestinal bleeding	16 (3.0)	10 (3.8)	6 (2.2)	0.285
Intra-abdominal infection	10 (1.9)	5 (1.9)	5 (1.9)	0.967
Anastomosis leakage	17 (3.2)	4 (1.5)	13 (4.8)	0.030
Other	3 (0.6)	1 (0.4)	2 (0.7)	0.578
**Re-admission**	20 (3.7)	14 (5.3)	6 (2.2)	0.060
**Postoperative mortality**	5 (0.9)	2 (0.8)	3 (1.1)	0.674
**Clavien-Dindo ≥3**	21 (3.9)	6 (2.3)	15 (5.5)	0.052
**Conversion**			7 (2.6)	

Data are presented as *n* (%) unless otherwise indicated; the *p*-value was determined by the chi-square test for multiple comparisons and the Mann-Whitney *U* test for continuous variable (operative time, blood loss, and length of postoperative hospital stay).

### Pathological findings

The pathological parameters recorded for CRC are presented in Table 3. Regarding the T and N stages, the cancers in the open group were at much higher stages than those in the laparoscopic group. The number of harvested lymph nodes did not significantly differ between the two groups (30.9 ± 18.3 vs. 30.2 ± 15.3, *p* = 0.981). Additionally, there were no significant differences in the histological type and histological grade of the tumours between the two groups.

**Table 3:** Pathological characteristics of the study population.

Pathological characteristic	All patients	Open	Laparoscopic	*p*-value
**Number of patients (%)**	537 (100)	265 (49.3)	272 (50.7)	
**Tumour size (cm)**	4.76 ± 6.14	4.69 ± 2.17	4.83 ± 8.37	0.003
**T stage**				0.001
Tis	8 (1.5)	3 (1.1)	5 (1.8)	
T1	68 (12.7)	23 (8.7)	45 (16.5)	
T2	72 (13.4)	25 (9.4)	47 (17.3)	
T3	297 (55.3)	160 (60.4)	137 (50.4)	
T4	92 (17.1)	55 (20.4)	37 (14.0)	
**Number of resected lymph nodes**	30.5 ± 16.8	30.9 ± 18.3	30.2 ± 15.3	0.981
**N stage**				0.028
N0	300 (55.9)	133 (50.2)	167 (61.4)	
N1	135 (25.1)	73 (27.5)	62 (22.8)	
N2	102 (19.0)	59 (22.3)	43 (15.8)	
**Histology**				0.761
Adenocarcinoma	509 (94.8)	253 (95.5)	256 (94.1)	
Signet ring cell	5 (0.9)	2 (0.8)	3 (1.1)	
Mucinous	23 (4.3)	10 (3.8)	13 (4.8)	
**Grade**				0.055
Well	74 (13.8)	29 (10.9)	45 (16.5)	
Moderate	410 (76.4)	214 (80.8)	196 (72.1)	
Poor	47 (8.8)	21 (7.9)	26 (9.6)	

Data are presented as *n* (%) unless otherwise indicated; the *p*-value was determined by the chi-square test for multiple comparisons and the Mann-Whitney *U* test for continuous variable (tumour size and number of resected lymph nodes).

### Clinical factors related to morbidity

Table 4 shows results of the multivariable analysis of the clinical factors influencing postoperative morbidity. In univariate analysis, patients with a high BMI, pulmonary disease, abnormal serum CEA level, hypoalbuminaemia, prolonged operative time, and increased amount of blood loss were significantly correlated with postoperative morbidity. Performing colorectal resection for obese patients using the laparoscopic method had no correlation with postoperative morbidity. In multivariate analysis, the independent predictors of worse outcome were hypoalbuminaemia [odds ratio (OR): 5.050, *p* < 0.001] and prolonged operative time (OR: 1.004, *p* < 0.001).

**Table 4:** Results of multivariable analysis of clinical factors influencing postoperative morbidity.

Variable	Univariate analysis		Multivariate analysis	
	Odds ratio (95% CI)	*p*-value	Odds ratio (95% CI)	*p*-value
Age ≥65 years	1.332 (0.835–2.124)	0.229		
Sex (M vs. F)	1.061 (0.665–1.692)	0.804		
Previous abdominal surgery	0.944 (0.529–1.682)	0.844		
BMI	1.077 (0.995–1.166)	0.068	1.059 (0.972–1.153)	0.189
Body weight loss	0.753 (0.309–1.831)	0.531		
Physical activity	0.658 (0.289–1.499)	0.319		
Hypertension	0.735 (0.456–1.185)	0.207		
Cardiac disease	1.085 (0.707–1.665)	0.708		
Cerebrovascular accident	1.029 (0.344–3.076)	0.960		
Pulmonary disease	2.279 (0.969–5.361)	0.059	1.519 (0.593–3.893)	0.384
Diabetes mellitus	1.403 (0.867–2.268)	0.168		
Chronic hepatitis	0.935 (0.315–2.776)	0.903		
Liver cirrhosis	3.319 (0.778–14.158)	0.105		
Thyroid disease	1.817 (0.360–9.159)	0.469		
Renal insufficiency	1.590 (0.864–2.928)	0.136		
Charlson Comorbidity Index	1.052 (0.941–1.177)	0.372		
Neoadjuvant therapy	1.358 (0.652–2.830)	0.414		
CEA level >5 ng/ml	1.585 (0.965–2.603)	0.069	1.430 (0.844–2.422)	0.183
Hb <10 g/dl	1.519 (0.751–3.076)	0.244		
Absolute lymphocyte count <1,500	1.280 (0.754–2.173)	0.361		
Albumin <3.5 g/dl	6.097 (2.710–13.698)	<0.001	5.050 (2.155–11.904)	<0.001
Emergency surgery	1.080 (0.125–9.359)	0.945		
Combined surgery	1.120 (0.479–2.619)	0.793		
Stage (0 vs. 1 vs. 2 vs. 3 vs. 4)	1.000 (0.791–1.265)	0.997		
Tumour size	1.002 (0.967–1.039)	0.913		
T (1 vs. 2 vs. 3 vs. 4)	1.182 (0.910–1.534)	0.211		
N (0 vs. 1 vs. 2)	0.976 (0.724–1.317)	0.876		
M (0 vs. 1)	0.979 (0.910–1.053)	0.564		
Operative time	1.005 (1.003–1.007)	<0.001	1.004 (1.002–1.007)	<0.001
Laparoscopic technique	1.026 (0.644–1.634)	0.914		
Blood loss	1.002 (1.001–1.003)	<0.001	1.001 (1.000–1.002)	0.166

The *p*-value was determined by binary logistic regression for univariate and multivariate analyses. CI, confidence interval, BMI, body mass index, CEA, carcinoembryonic antigen, Hb, haemoglobin; vs., versus; M, male, F, female.

## Discussion

The present study showed that even though the time of laparoscopic resection for CRC was longer than that of open surgery, the laparoscopic group had less blood loss and a shorter postoperative hospital stay when compared with the open group. The study also revealed similar overall postoperative morbidity and mortality rates between the open and laparoscopic groups for CRC patients with generalized obesity.

Previous randomised controlled trials (RCTs) have already confirmed that laparoscopic surgery is equivalent to conventional open surgery in patients with colon and rectal cancer (10, 21). However, these RCTs did not evaluate CRC patients with generalized obesity as patients were excluded from the trial if their BMI was ≥30 kg/m^2^. After reviewing the previous literature, the issue of obesity and laparoscopic surgery was focused on comparing obese and non-obese patients with CRC (22–25). In order to reflect the application of laparoscopic surgery in real-world treatment experience for CRC patients with generalized obesity, we compared the laparoscopic and open surgery outcomes in only a specific population of patients with generalized obesity. To the best of our knowledge, this is the first study to only focus on CRC patients with a BMI ≥30 kg/m^2^ and collect the largest sample of CRC patients with generalized obesity from a single academic medical centre.

A higher incidence of perioperative morbidity and mortality, including myocardial infarction, thrombotic complications, nerve injuries, respiratory diseases, wound infection, and urinary infection, was noted in the generalized obesity population when they received the operation (4). Hence, comprehensive preoperative evaluation, perioperative precautions, and postoperative care for patients with generalized obesity are necessary (26). Compared to non-obese CRC patients, those with generalized obesity are considered to be associated with poor perioperative outcomes in colorectal surgery, including a longer operative time, greater blood loss, reduced number of harvested lymph nodes, higher conversion rate, and more postoperative complications (27, 28).

In our study, although CRC patients with generalized obesity who received laparoscopic surgery had a higher percentage of diabetes mellitus and liver cirrhosis, their blood loss was significantly less and postoperative hospital stay was significantly shorter than those of patients who received open surgery. This result means that the CRC patients with generalized obesity who underwent laparoscopic surgery were comparable to the patients of previous studies who underwent laparoscopic colorectal surgery and also had a shorter length of hospital stay and less blood loss (29–31). We also revealed that the number of harvested lymph nodes did not differ between the open and laparoscopic surgery groups in CRC patients with generalized obesity. We expect that laparoscopic surgery in CRC patients with generalized obesity should have oncological outcomes similar to those of open surgery after long-term follow-up. The CRC patients with generalized obesity who underwent laparoscopic surgery or open surgery also had similar short-term outcomes in terms of the incidences of total postoperative morbidity and mortality. After multivariate analysis was performed, the surgical method (open or laparoscopic surgery) was found to not be an independent risk factor for postoperative morbidity and mortality in CRC patients with generalized obesity.

Lai et al. that hypoalbuminaemia colon cancer patients had a higher rate of postoperative mortality and morbidity, including complications related to wounds, lungs, the urinary system, and anastomosis (32). Prolonged operative time was a significant risk factor for surgical site infection in colorectal cancer surgery (33). And we got the same result in the multivariate analysis that hypoalbuminaemia and prolonged operative time were two independent predictors of postoperative complication in CRC patients with generalized obesity. The result indicated that the obesity population had similar risk factors of postoperative complication with the general population.

A conversion rate of 2.6% in CRC patients with generalized obesity who underwent laparoscopic surgery was another highlight of our study. After reviewing several RCTs, including the CLASICC, COST, and COLOR II trials in the last decade, the general conversion rates of laparoscopic colorectal surgery ranged from 16% to 29% (8, 9, 21). Generalized obesity was considered to be associated with a high conversion rate in a previous study (16). After advancements in techniques and improvements in equipment for the laparoscopic approach, our institution had already conquered the learning curve effect over these decades and could provide advanced laparoscopic surgery to patients with CRC, including the population with generalized obesity.

For patients with a high BMI, because of visceral and abdominal wall adiposity, the restricted operative space and difficulty of the operative technique prolonged the operative time in open or laparoscopic surgery (34, 35). In our study, although the CRC patients with generalized obesity who underwent laparoscopic surgery had a lower percentage of previous abdominal surgery and fewer advanced tumour stages, the operative time was still longer than that in patients who underwent open surgery. Previous meta-analysis studies also confirmed that laparoscopic surgery may require a longer operative time than open surgery in the general population (36). Although the surgery time was prolonged in the laparoscopic group, our results showed that there was no increase in postoperative morbidity and mortality rates, implying that laparoscopic surgery could be a safe method for patients with obesity.

A statistically higher anastomotic leakage rate was noted in the laparoscopic surgery group (1.5% vs. 4.8%, *p* = 0.030) of CRC patients with generalized obesity. In our database, the diverting stoma rate was a higher tendency in the open group than in the laparoscopic group (14.2% vs. 9.4%, *p* = 0.096), as diversion of the stoma can protect the intestinal anastomosis in the abdominal cavity. The percentages of abdominal perineal resection and the Hartmann procedure for end stoma creation were higher in the open group than in the laparoscopic group (7.1% vs. 2.2%, *p* = 0.007) because avoidance of intestinal anastomosis was necessary during the operation. A diverting stoma or end stoma may mask the symptoms of leakage, resulting in an incorrect estimation of the leakage rate. The other reason is that in the early era of laparoscopic application, the instruments were not very advanced. The old-type stapler for anastomosis had a higher rate of anastomosis insufficiency, and the blood supply of the anastomosis stump may not be easy to confirm through laparoscopy in the past. Compared to other studies, the incidence of anastomotic leakage was between 3% and 28% in CRC surgery (37). The anastomotic leak rate in laparoscopic group was 4.8% and was within the acceptable range. If the diverting stoma creation were indicated in the laparoscopic group for treatment of anastomosis leakage, the procedure would be performed through laparoscopy without extending the laparotomy wound. Because of the abdominal wall adiposity in patients with generalized obesity, diversion ostomy creation after the leakage episode was difficult in the generalized obesity population. In selected cases, conservative treatment with or without diverting stoma creation, adequate intrabdominal and pelvic irrigation through laparoscopy, or drainage abscess by image guide was the primary treatment in our hospital. In the laparoscopic group, patients who encountered anastomosis leakage and under adequate postoperative monitoring and care strategy, the mortality rate did not higher than in the open group (2 of 13 anastomosis leakage patients died in the laparoscopic group and 1 of 4 patients in the open group.).

There are a couple of limitations to our study. This retrospective study was conducted over a decade between January 2009 and December 2019. Although the technique of laparoscopic surgery has seen a booming improvement in our medical institution in recent decades, the selection bias of choosing laparoscopic or open surgery is subjective to surgeons’ experience and patients’ performance. Also, there are some confounding and selection biases to the limitation, like DM, liver cirrhosis, stoma rate, and stage distribution. The differences between the open and laparoscopic patients may interfere with the results of the outcome. However, the multivariable analysis revealed that the operation method did not correlate with postoperative morbidity. In the future, an RCT to evaluate the effect of laparoscopic surgery in CRC patients with generalized obesity may be required.

## Conclusion

Our study demonstrates that laparoscopic surgery is a feasible procedure for CRC patients with generalized obesity in the short-term outcome. Except for the increased risk of anastomotic leakage, we revealed that the laparoscopic approach did not increase the incidence of postoperative morbidity and mortality in CRC patients with generalized obesity. Although the average operative time is longer in laparoscopic surgery than in open surgery, the laparoscopic approach can reduce blood loss during surgery, decrease the postoperative length of hospital stay, and obtain a similar number of harvested lymph nodes compared to the open approach in CRC patients with generalized obesity. The low conversion rate in CRC patients with generalized obesity after advancement in the technique was another highlight of the laparoscopic approach. Under the acceptably low complication rate, laparoscopic surgery could become a feasible procedure for treating CRC in patients with generalized obesity after excluding the injustice of the biases.

## Data Availability

The raw data supporting the conclusions of this article will be made available by the authors, without undue reservation.
